# Patients harboring uncommon EGFR exon 19 deletion-insertion mutations respond well to first-generation EGFR inhibitors and osimeritinib upon acquisition of T790M

**DOI:** 10.1186/s12885-021-08942-x

**Published:** 2021-11-13

**Authors:** Yurong Wang, Ruipan Zheng, Peizhu Hu, Ziheng Zhang, Shujing Shen, Xingya Li

**Affiliations:** 1 Department of Medical Oncology, The First Affiliated Hospital of Zhengzhou University, Jianshe East Road 1, 450052 Zhengzhou, Henan People’s Republic of China; 2 Department of Radiotherapy, The First Affiliated Hospital of Zhengzhou University, Jianshe East Road 1, 450052 Zhengzhou, Henan People’s Republic of China; 3 Department of Pathology, The First Affiliated Hospital of Zhengzhou University, Jianshe East Road 1, 450052 Zhengzhou, Henan People’s Republic of China

**Keywords:** EGFR exon 19 deletion-insertion, EGFR TKI, Clinical outcomes, Resistance mechanism, NSCLC

## Abstract

**Background:**

In the existing next generation sequencing (NGS) system, epidermal growth factor receptor (EGFR) exon 19 deletion-insertion (19delins) is still interpreted into the category of EGFR exon 19 deletion (19del). However, the controversy exists whether the two mutation types have the similar responses and resistant mechanisms to first-generation EGFR tyrosine kinase inhibitor (TKI) in non-small cell lung cancer (NSCLC) patients.

**Methods:**

We successively and retrospectively reviewed the NGS data of 3054 patients diagnosed as advanced NSCLC from November 2017 to September 2020. Finally, 41 patients with EGFR 19delins mutation and 41 patients with EGFR 19del mutation who received first-generation EGFR TKIs as first-line therapy were included in the study.

**Results:**

A total of 17 genotypes were identified in this study, including L747_P753delinsS (10/41), L747_A750delinsP (9/41), L747_T751delinsP (6/41) and E746_S752delinsV (3/41). Under the same baseline characteristics, the population of EGFR 19delins respond well to first line EGFR TKIs as well as those of EGFR 19del, with little difference in median progression-free survival (mPFS): 10.4 months vs. 13.1 months, *p* = 0.1076). Interestingly, patients with L747_T751delinsP seem to have a better mPFS than others (18.7 months vs. 13.1 months, *p* = 0.035). After the disease progression, both EGFR 19delins and EGFR 19del had similar rates of developing EGFR T790M mutation resistance (45.8% vs. 57.8%), and those receiving osimeritinib as second-line treatment obtain the similar survival benefits (mPFS: 12.0 months vs. 12.2 months (*p* = 0.97).

**Conclusions:**

This retrospective cohort study furnish the evidence that therapeutic responses and survival of untreated NSCLC population with EGFR 19delins mutation are equal to those with common EGFR 19del mutation after administration of EGFR TKIs therapy.

**Supplementary Information:**

The online version contains supplementary material available at 10.1186/s12885-021-08942-x.

## Key points

With the increase use of NGS in clinical routine practice, more and more rare gene mutations have been identified.

In the existing NGS report system, EGFR 19delins is still interpreted into the category of deletion in exon 19. However, the controversy exists whether the two population have the similar responses and resistant mechanisms to EGFR TKIs.

This retrospective cohort study furnish the evidence that therapeutic responses and survival of untreated NSCLC population with EGFR 19delins mutation are equal to those carrying common EGFR 19del mutation with first-generation EGFR TKI therapy.

At progression to first-generation EGFR TKIs, the resistance mechanism of patients with uncommon EGFR 19delins mutation have been demonstrated, as well as the effects of second-line osimeritinib treatment after the acquisition of T790M mutation.

## Background

Non-small cell lung cancer (NSCLC), accounts for 85–90% of all lung cancers, is the leading cause of cancer-related mortality in China and worldwide [1]. Epidermal growth factor receptor (EGFR) mutations are one of the most frequent oncogenic driver events in NSCLC. Among them, exon 19 deletions and the L858R point mutation comprise up to 90% EGFR mutations and confer hypersensitivity to EGFR tyrosine kinase inhibitors (TKIs), called classical mutations [2]. With the increasing use of next-generation sequencing (NGS) in clinical practice, more low frequency mutations have been identified, such as EGFR 19delins variants [3]. However, our understanding of their biology and clinical response to EGFR TKIs is poorer than that of classical mutations.

The clinical responses of EGFR 19delins to EGFR TKIs are reported to be different. Truini et al. proved that patients with L747_A750delinsP had significantly worse PFS and OS than those with common 19del and L747_P753delinsS [4]. In addition, some case reports revealed EGFR 19delins confer primary resistance to gefitinb or erlotinib [5–7]. On the contrary, some researchers reported that patients with EGFR 19delins mutation, such as L747P, had favorable outcomes upon EGFR-TKI therapy [8, 9]. However, the above results are based on limited cases and inconsistent. Furthermore, the resistance mechanisms to first-generation EGFR-TKIs are not studied in the population of EGFR 19delins. The study from Peng et al. has shown that first-line EGFR TKIs bring better survival benefits to patients with EGFR 19delins than those with EGFR 19del, but once upon the acquisition of T790M resistance mutation, the outcomes of EGFR 19delins group are poor [10]. Nevertheless, different from their findings, our research demonstrated that patients carrying EGFR 19delins mutation can also benefit from first-generation EGFR TKIs (gefitinib and Icotinib) equally to those carrying EGFR 19del mutation. Besides, we also analyzed the resistance mechanism of EGFR 19delins to first-generation TKIs and the outcomes of those receiving second-line osimeritinib upon acquisition of T790M mutation.

## Methods

### Patients

**Fig. 1:**
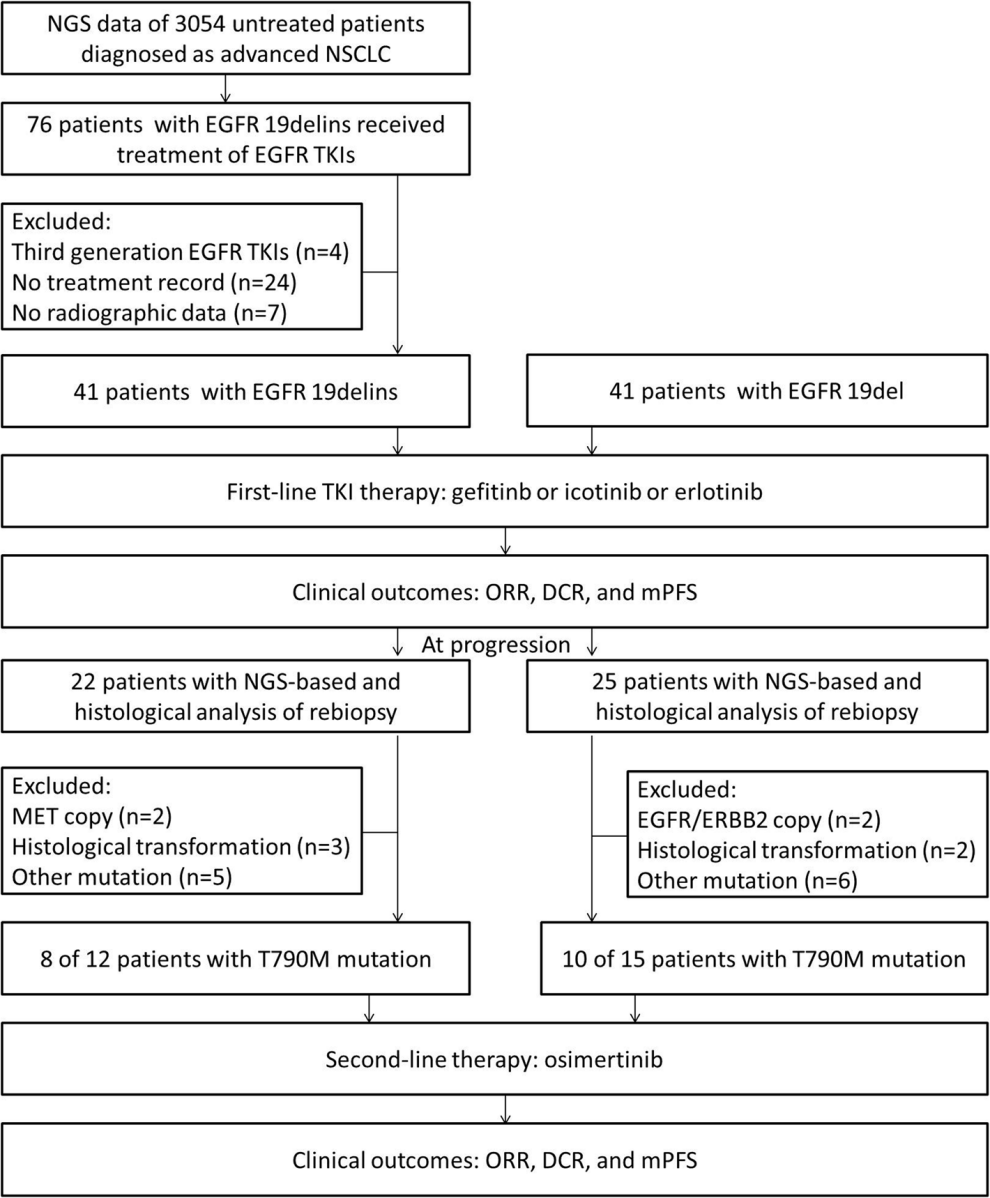
Flow chart of the study design. 3054 treatment-naïve patients with advanced NSCLC performing NGS molecular test in our hospital were successively screened. 41 patients with EGFR 19delins mutation met the inclusion criteria, and then 41 patients with EGFR 19del mutation were enrolled successively at random, receiving first-generation EGFR TKI gefitinib or icotinib or erlotinib as first-line therapy. At progression, the resistance mechanisms were analyzed. And the PFS and response rates after second-line osimertinib were also evaluated

The NGS data of 3054 patients diagnosed as advanced NSCLC were retrospectively and successively screened between November 2017 and September 2020. And 41 treatment-naïve patients at diagnosis with EGFR 19delins mutation met the inclusion criteria, and then 41 untreated patients with EGFR 19del mutation were enrolled successively at random in this study. All these patients were given gefitinib (250 mg once daily) or icotinib (125 mg three times a day) or (erlotinib 150 mg once daily) orally as first-line therapy. Clinical outcomes of these patients were evaluated (Fig. 1). The inclusion criteria for patients in the study were similar to the previous study [10], including: NSCLC confirmed by immunohistochemistry, stage IV disease according to the 8th American Joint Committee on Cancer Staging System, age beyond 18 years, first-line standard EGFR TKI therapy, genotypes of EGFR 19delins or EGFR 19del identified by NGS, radiographic images data available at baseline and progression per Response Evaluation Criteria in Solid Tumors, version 1.1. Patients with baseline EGFR T790M mutation and other systematic antitumor treatment were excluded. The Ethics Committee of First Affiliated Hospital of Zhengzhou University approved this retrospective analysis and waived informed consent.

### DNA extraction and routine driver gene testing

DNA extraction and sequencing methods were described as the previously protocol [10]. Briefly, a minimum of 20 ng of DNA extracted from tumor tissues or liquid samples (such as, pleural effusion and blood) is required for NGS library construction. The molecular test was performed on Illumina HiSeq 4000 (Illumina, San Diego, CA) using commercially hybrid capture-based NGS assays including 8, 14 or 56 gene panels (GeneseeqOne, Nanjing Geneseeq Technology Inc., China; Burning Rock Biotech Ltd., Guangzhou, China) with sequencing depth of 1000× in tissues and 5000× in liquid sample at least. All the above panels can at least identify the below routine driver genes: EGFR, KRAS, ALK, ROS1, RET, MET, ERBB2 and BRAF. Finally, the NGS data were mapped to the human genome 19 for accurate identification of mutations. The adapters in FASTQ files of NGS data were cleaned using Trimmomatic-0.36 software [11]. Then, Single nucleotide variants (SNVs) and short insertions/deletions (indels) were identified using VarScan2 2.3.9 with minimum variant allele frequency (VAF) threshold set at 0.01 and *p*-value threshold for calling variants set at 0.05 to generate Variant Call Format files. All SNVs/indels were annotated with ANNOVAR, and each SNV/indel was manually checked with the Integrative Genomics Viewer. Copy number variations were identified using ADTEx 1.0.4. Genomic fusions were identified by FACTERA software [12].

For the first biopsy, pleural sediment embedding was selected for subsequent sequencing in patients with huge malignant pleural effusion, with small lung lesions and no other superficial metastases available for biopsy or with high risk of tissue biopsy. And the time points of NGS for first biopsy ranged from November 2017 to August 2020 in EGFR 19delins cohort and February 2018 to July 2020 in EGFR 19del cohort. And there were two cases (April 2018 and March 2018) and one case (June 2018) using pleural sediment embedding as samples in EGFR 19delins cohort and EGFR 19del cohort, respectively. And the rest cases were tissue samples. For the second biopsy, tissue biopsy and blood biopsy were adopted for NGS from August 2018 to June 2020 in EGFR 19delins group and April 2019 to August 2020 in EGFR 19del group. In EGFR 19delins group, three cases performed blood biopsy (December 2019, April 2020, and July 2020), while one case (January 2019) in EGFR 19del group. And the rest cases were tissue samples.

### Evaluation

The clinical characteristics were reviewed and collected from the medical records, including age, sex, pathological information, NGS molecular profile, treatment and radiographic data, and so on. The best changes of tumor from baseline, response rates, and progression-free survival (PFS) were analyzed according to the radiographic images.

### Statistical analysis

The baseline data was compared by chi-square test or fisher-exact test for some variables when chi-square test was not appropriate. Propensity score (PS) correction method was used for controlling confounding factors, such as age, gender, ECoG score, T stage, N stage, brain metastases, first line TKIs. The survival analysis was performed using the Kaplan-Meier method with the statistical packages R (The R Foundation; http://www.r-project.org; version 3.4.3) and Empower (R) (www.empowerstats.com, X&Y solutions, inc. Boston, Massachusetts) [13]. Two sided *p* values less than 0.05 were considered statistically significant.

## Results

### Patients’ baseline characteristics

Among 3054 patients diagnosed as advanced NSCLC, 41 treatment-naive patients with EGFR 19delins mutation were enrolled in this study, with 41 patients carrying EGFR 19del mutation as control cohort. Patients with EGFR 19delins were mostly women (24/41), non-smokers (34/41), stage IV disease (41/41) and adenocarcinoma histology (40/41). The clinical baseline characteristics of the cohorts of uncommon EGFR 19delins and EGFR 19del were summarized in Table 1.

**Table 1:** Baseline characteristics of the NSCLC patients with EGFR 19delins or EGFR 19del

	Characteristics	19delins (*n* = 41)	19del (*n* = 41)	*P* value
Age, years	Median (range)	60 (30–75)	60 (34–76)	0.638
Sex	Male	17	11	0.162
	Female	24	30	
Smoking status	Never	34	34	1.000
	Smoker	7	7	
Histological type	Adenocarcinoma	40	41	1.000
	Squamous carcinoma	1	0	
TNM stage	IIIB	0	0	1.000
	IV	41	41	
ECOG PFS score	0 or 1	34	31	0.414
	2	7	10	
Brain metastases	No	25	27	0.647
	Yes	16	14	
EGFR 19delins variants	p.L747_P753delinsS	10		
	p.L747_A750delinsP	9		
	p.L747_T751delinsP	6		
	p.E746_S752delinsV	3		
	Other EGFR 19delins	13		
EGFR 19del variants	p.E746_A750del		36	
	p.L747_S752del		3	
	P.L747_T751del		2	
First-line treatment	Gefitinib	24	25	0.973
	Icotinib	15	14	
	Erlotinib	2	2	

19delins, EGFR exon 19 deletion-insertion; 19del, EGFR exon 19 deletion; *ECOG PS* Eastern Cooperative Oncology Group performance status

### Baseline mutation profile

At baseline, 8-gene panel (26.8%, 11/41), 14-gene panel (36.6%, 15/41), and 56-gene panel (36.6%, 15/41) were using for NGS profiling in EGFR 19delins cohort. Among the 41 patients with EGFR 19del, the NGS profiling was performed by 8-gene panel (24.4%, 10/41), 14-gene panel (41.5%, 17/41), and 56-gene panel (31.1%, 14/41). In our study, patients concurrent with other actionable oncogenic driver mutations were excluded from both cohorts. EGFR amplification and TP53 mutation were the most frequent concurrent mutations identified with 17.1% (7/41) and 9.8% (4/41) in the EGFR 19delins cohort, and 17.1% (7/41) and 17.1% (7/41) in EGFR 19del cohort. There is need to note that only the 56-gene panel is capable of detecting TP53 mutations. And TP53 co-mutation had no significant effect on the survival of EGFR 19delins and EGFR 19del cohort (*p* > 0.05) (Fig. S1). The ratio of sequencing panels and concurrent mutations were basically the same in the two groups.

### The variants detected in uncommon EGFR 19delins cohort

**Fig. 2:**
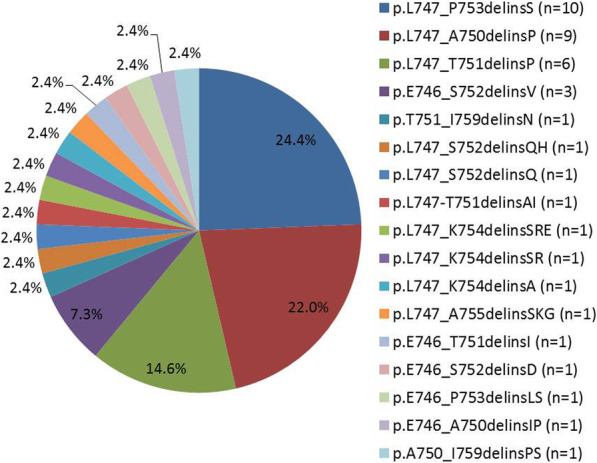
Pie charts illustrating the distribution of EGFR 19delins variants among 41 patients

There were 17 different mutation types were identified in the EGFR 19delins cohort (Fig. 2). Among them, three were new detected and not published in previous literatures and somatic mutation database (Catalogue of Somatic Mutations in Cancer, COSMIC), such as L747_K754delinsSR, A750_I759delinsPS, and L747-T751delinsAI. The most frequent mutation types in the EGFR 19delins cohort were L747_P753delinsS (*n* = 10, 24.4%), L747_A750delinsP (*n* = 9, 22.0%), L747_T751delinsP (*n* = 6, 14.6%), and E746_S752delinsV (*n* = 3, 7.3%, Fig. 2B). Distribution of EGFR 19delins nutation types have been illustrated using pie charts figure. And EGFR amplification was the most common concurrent mutation in both uncommon EGFR 19delins and common EGFR 19del groups, with equal percentage of 17.1% (7/41). The VAF of the EGFR 19del and EGFR 19delins ranges from 0.53% to 86.4% and 0.08% to 90.8%, respectively (Table S1).

### The clinical effects of first-line EGFR inhibitors

The effects of first-line EGFR TKI treatment in the patients with EGFR 19delins and EGFR 19del were evaluated. The clinical baseline characteristics of the two cohorts were similar (Table 1). The patients with EGFR 19delins were given gefitinib (*n* = 24, 58.5%), icotinib (*n* = 15, 36.6%), and erlotinib (n = 2, 4.9%), respectively. Similarly, the patients with EGFR 19del were given gefitinib (*n* = 25, 61.0%), icotinib (*n* = 14, 34.1%), and erlotinib (n = 2, 4.9%), respectively.

**Fig. 3:**
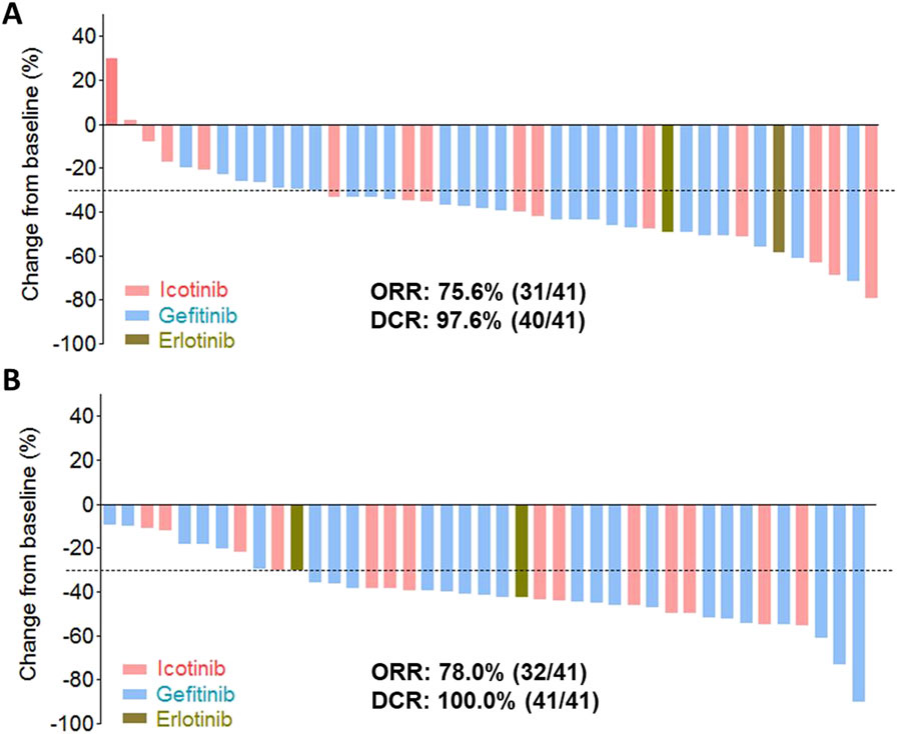
Best changes from baseline in tumor-size since treatment of first-generation EGFR TKIs in the cohorts of EGFR 19delins (**A**) and EGFR 19del (**B**)

The best changes of tumor size from baseline after first-line TKI treatment in the two cohorts were estimated (Fig. 3A and B). The objective response rate (ORR) was similar between the two cohorts (EGFR 19delins vs. EGFR 19del: ORR 75.6% vs. 78.0%; DCR 97.6% vs. 100%). Among the patients with EGFR 19delins, 31 patients achieved partial response (PR; 75.6%), 9 patients had stable disease (SD; 22.0%) and one patient had progressive disease (PD, 2.4%) after first-line EGFR TKI. Likewise, in the patients carrying EGFR 19del, the rates of PR, SD, and PD were 78.0% (32/41), 22.0% (9/41) and 0.

**Fig. 4:**
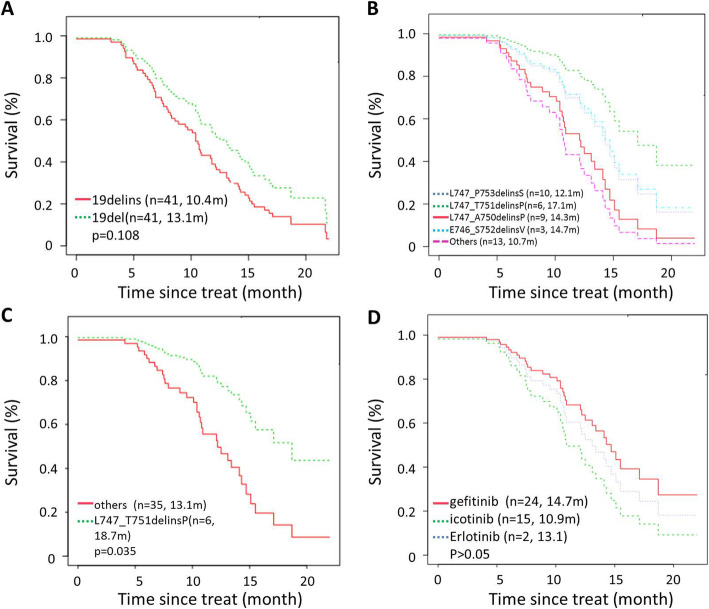
Patients harboring rare EGFR 19delins mutation obtained similar survival benefits from first-generation EGFR TKIs to those with EGFR 19del mutation. Kaplan-Meier curves were drawn for comparing the PFS between patients with EGFR 19delins and EGFR 19del (**A**), PFS among patients with different variants in EGFR 19delins cohort (**B** and **C**), and PFS between patients receiving different EGFR TKIs in EGFR 19delins cohort (**D**)

PS correction method was used for controlling confounding factors, such as age, gender, ECoG score, T stage, N stage, brain metastases, first line TKIs (the binary logistic regression model: PS = -1.345 + 0.569 × sex-0.015 × age+ 0.411 × ECOG score- 0.305 × T stagte+ 1.126 × N stage-0.216 × brain metastases-0.020 × first line TKIs). The distribution of PS between the cohorts of EGFR 19delins and EGFR 19del were consistent (the population pyramid is symmetric), mainly distributed between 0.40 and 0.60 (Fig. S2). Then survival analysis was performed under the adjustment of PS. The median PFS (mPFS) was 10.4 month in patients with EGFR 19delins, similar to 13.1 months in those with EGFR 19 del (HR: 0.6527; 95% CI: 0.3885-1.0983; *p* = 0.108; Fig. 4A). Interestingly, the outcomes of patients with different EGFR 19delins mutation types are not exactly same. As shown in Fig. 4B and C, patients harboring L747_T751delinsP had a longer mPFS than those harboring L747_P753delinsS, L747_A750delinsP, and other variants (18.7 months vs 13.1 months, HR: 0.3318; 95% CI: 0.1193-0.9232; *p* = 0.035). There is no significant difference between patients with L747_A750delinsP and L747_T751delinsP (Fig. 4B). Despite of limited cases, we also attempted to analyze the effects of three first-generation EGFR TKIs in patients with EGFR 19delins. And no significant difference was found among the groups of gefitinib, icotinib, and erlotinb with 14.7 months, 10.9 months, and 13.1 months (*p* > 0.05; Fig. 4D). Besides, no serious adverse events (grade 3/4) were reported by the two cohorts.

### The mechanisms of resistance to first-generation EGFR TKIs

**Fig. 5:**
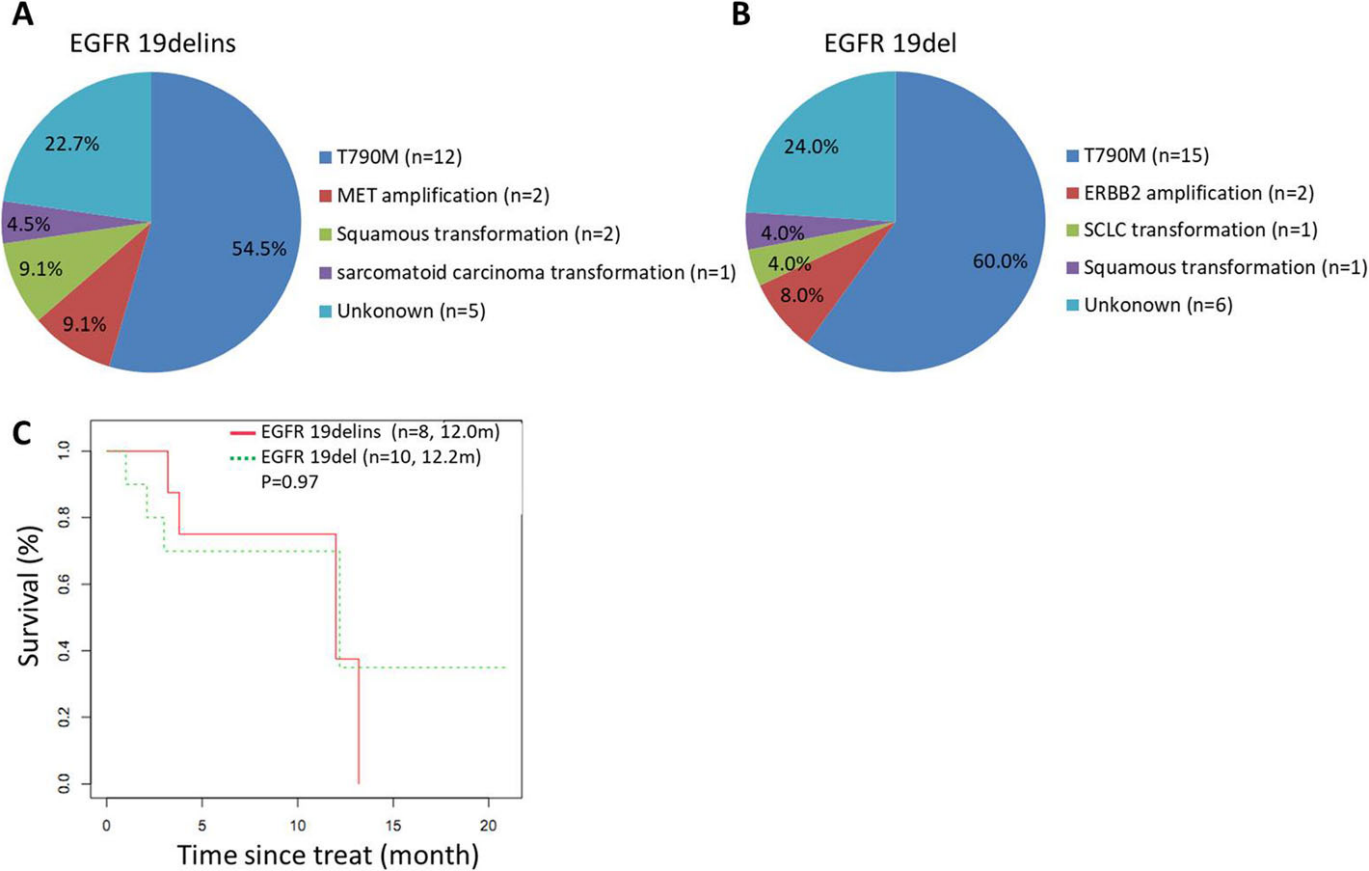
Potential mechanisms resistant to first-generation EGFR inhibitors and survival benefits of osimeritinib for patients developing T790M mutation. Resistance reasons to first-generation EGFR TKIs were described in the cohorts of EGFR 19delins (**A**) and EGFR 19del (**B**). **C** Kaplan-Meier curves comparing the PFS between patients with EGFR 19delins and those with EGFR 19del after administration of osimeritinib as second-line therapy when developing EGFR T790M resistance

After administration of first-generation EGFR inhibitors, 34 patients carrying EGFR 19delins and 32 patients carrying EGFR 19del had PD. At progression, 64.7% (22/34) in EGFR 19delins cohort and 78.1% (25/32) in EGFR 19del cohort performed re-biopsies and NGS test. And EGFR T790M was the main resistance mechanism in the two cohorts without statistical significance, *p =* 0.706 (EGFR 19delins vs. EGFR 19del: 54.5% (12/22) vs. 60.0% (15/25). The resistance mutation was not identified in 22.7% (5/22) and 24.0% (6/25) of the patients with EGFR 19delins and EGFR 19del, respectively. Besides, one case had sarcomatoid carcinoma transformation and two cases had squamous transformation from adenocarcinoma in EGFR 19delins cohort. Comparatively, in EGFR 19del cohort, there was one each case had small-cell lung cancer transformation and squamous transformation from adenocarcinoma. MET amplification was acquired in (2/22) EGFR 19delins group and ERBB2 amplification was acquired in 1/25 of EGFR 19del cohort (Fig. 5A and B).

### Clinical benefits of osimeritinib for patients developing T790M mutation

Whether patients with EGFR 19delins respond well to third-generation EGFR TKIs after acquiring EGFR T790M resistance to first generation EGFR TKIs? To figure out that, we analyzed the clinical benefits of osimeritinib as second-line therapy for patients with EGFR 19delins (*n* = 8) and those with EGFR 19del (*n* = 10) after developing T790M mutation. The therapeutic responses were similar in both EGFR 19delins and EGFR 19del groups: ORR 62.5% vs 30.0%, *p* = 0.34; DCR: 100.0% vs 80.0%, *p* = 0.48 (Table 2). Also, no significant difference were observed in mPFS between EGFR 19delins and EGFR 19del groups (mPFS: 12 months vs. 12.2 months; Log-rank test, *p* = 0.97; Fig. 5C).

**Table 2:** Clinical responses of third-generation EGFR-TKI as second line therapy in NSCLC patients with EGFR 19delins and EGFR 19del

	19delins (n = 8)	19del (n = 10)	*P* value
ORR, n (%)	5 (62.5)	3 (30.0)	0.34
DCR, n (%)	8 (100.0)	8 (80.0)	0.48
Best objective response, n (%)			
CR	0	0	
PR	5 (62.5)	3 (30.0)	
SD	3 (37.5)	5 (50.0)	
PD	0	2 (20.0)	

*CR* Complete response, *PR* Partial response, *SD* Stable disease, *PD* Progressive disease, *ORR* Objective response rate, *DCR* Disease control rate

## Discussion

The present study analyzed the variants and treatment outcomes to first-generation EGFR TKIs as first-line therapy in Chinese patients with rare EGFR 19delins mutation. After progression, the resistant mechanisms to first-generation EGFR TKIs and the effects of osimeritinib on patients with EGFR T790M mutation have also been investigated. A total of 17 mutation types of uncommon EGFR 19delins are identified and three are previously unpublished in any literatures and somatic mutation database.

EGFR 19del is the most common variant type of EGFR mutation in NSCLC [14]. Clinically, EGFR genotyping methods include amplification refractory mutation system (ARMS)-polymerase chain reaction (PCR), sanger sequencing, and NGS. Despite the most common use in clinical practice, ARMS-PCR are not capable of covering the entire spectrum of EGFR mutations based on the current mutation-specific PCR kits, therefore some potentially treatable mutations are inevitably missed by this approach [15–17]. Sanger sequencing can identify all mutations in a given gene region, however, false negatives still occur especially when tumor cells are insufficient. For this problem, NGS can profile all types of EGFR mutations and concurrent mutations with high sensitivity. To this end, NGS method was used for screening patients in our study. We have reviewed the NGS records of 3054 NSCLC untreated patients, and 41 objects with EGFR 19delins were enrolled, compared by 41 baseline-matchable objects with EGFR 19del mutation. The present study showed that the treatment responses and mPFS in the population with EGFR 19delins mutation are not worse than those with common EGFR 19del when receiving first-generation EGFR TKIs. Among the more frequent EGFR 19delins mutation types, L747_T751delinsP group seems to have a better mPFS after the administration of first-generation TKIs. At progression, the main resistant cause is EGFR T790M mutation both in EGFR 19delins and EGFR 19del groups. Moreover, the clinical outcomes including disease control rate and PFS are not significantly different.

Previous studies suggest that patients with some subtypes of EGFR 19delins mutation have poorer clinical outcomes than those with EGFR 19del [18]. For example, afatinib can inhibit phosphorylation of EGFR in both L747_P753delinsS and L747_A750delinsP subtypes, whereas erlotinib and osimeritinib only inhibit that in L747_P753delinsS mutation [18]. As for mechanisms underline, Simona Coco et al. consider that E746V-L747P (E746_L747delinsVP) variant confers resistance to gefitinib by mediating N terminal rearrangement of EGFR kinase domain, which leads to high affinity with ATP and EGFR activation [7]. However, another large scaled research has reported that patients with EGFR 19delins have longer PFS than that of common EGFR 19del population [10]. The mechanism may be that the insertion of one or more amino acids changes the conformation of EGFR kinase domain and enhances its binding to EGFR TKIs [10]. Interestingly, our data provide different views that the population with EGFR 19delins mutation has a good response to first-generation EGFR-TKIs and similar PFS outcomes to patients with common EGFR 19del mutation. And we speculate that only some deletion-insertion mutations cause conformational changes that affect their binding to EGFR TKIs, while others are not significantly different from the common deletion variants in exon 19 of EGFR gene on the therapeutic mechanisms of EGFR TKIs. For example, among the more frequent EGFR 19delins mutation types, L747_T751delinsP group has a better mPFS than L747_P753delinsS group. However, the exact mechanism underline still needs more preclinical experiments to illustrate. After disease progression on first-generation EGFR-TKIs, the acquired resistance mechanisms in this study are consistent with the published data [19], including EGFR T790M mutation, MET amplification, ERBB2 amplification, activation of EGFR downstream signaling, and histological transformation to small-cell or squamous cell carcinoma, even sarcomatoid carcinoma, and so on. Given that, tissue re-biopsy are strongly recommended for histological analysis and genotyping when disease progressed. In our research, EGFR T790M is still the major resistant mechanism for patients with EGFR 19delins after failure to first-generation EGFR TKIs. The efficacy of osimeritinib as second-line therapy is also similar between the groups of EGFR 19delins and EGFR 19del. Of note, it has been reported some certain variants of TP53 mutation may be one of the factors affecting the effects of targeted therapy and prognosis of patients with EGFR mutation [20]. While in the present study, no significant effects of TP53 co-mutation on the survival of patients with EGFR mutation (19delins or 19del) were observed. However, the results should be treated with caution due to the limited cases in this subgroup.

As a retrospective investigation, there are inevitably some limitations and confounding factors. In this regard, the cases are collected successively during a period and PS correction method was used for controlling confounding factors, such as age, gender, ECOG score, T stage, N stage, brain metastases, first line TKIs. In this study, NGS approach is adopted for screening patients due to possible missed detection of some mutations by PCR. However, PCR detection still accounts for a greater proportion in clinical practice currently, which means that a larger portion of the population was excluded. So whether our results are representative of the whole population with EGFR 19delins needs further verification in the future larger scaled and prospective trials.

## Conclusions

This study demonstrates that patients with rare EGFR 19delins have equal treatment responses and similar survival benefits to those with common EGFR 19del to first-line EGFR TKIs. Of note, patients with L747_T751delinsP mutation have a significant better survival outcome than those with other variants. At disease progression, EGFR T790M mutation is still the prevalent resistance mechanism of patients with EGFR 19delins and also sensitize to third-generation EGFR TKI, osimeritinib, similar to the population with EGFR 19del. Given that the most frequent and sensitive mutation in NSCLC, EGFR 19del (especially E746_A750del) is an indicator for good response to EGFR TKIs. Our results provide a proof that EGFR 19delins mutation also can serve as one good predictor for response to first-generation EGFR TKIs.

## Supplementary Information

**Additional file 1: Figure S1.** The effects of TP53 co-mutation on the survival of EGFR 19delins (A) and EGFR 19del (B) cohort.

**Additional file 2: Figure S2.** The distribution of propensity score (PS) between the cohorts of EGFR 19delins and EGFR 19del. X-axis of the population pyramid is the number of patients, and Y-axis is different propensity scores (0 ~ 1). It can be seen that propensity scores of EGFR 19delins (green) are similar to EGFR 19del (blue), mainly distributed between 0.4 and 0.6. The population pyramid is symmetric.

**Additional file 3: Table S1.** The VAF of EGFR 19delins mutation and EGFR 19del mutation, and the VAF of their T790M mutation acquired at progression.

## Data Availability

The datasets used and/or analyzed during the current study available from the corresponding author on reasonable request.

## Abbreviations

NGS: Next generation sequencing
EGFR: Epidermal growth factor receptor
19delins: 19 deletion-insertion
19del: 19 deletion
TKI: Tyrosine kinase inhibitor
NSCLC: Non-small cell lung cancer
mPFS: Median progression-free survival
PS: Propensity score
ARMS: Amplification refractory mutation system
PCR: Polymerase chain reaction
