# Supplementary figures and images for: Patients harboring uncommon EGFR exon 19 deletion-insertion mutations respond well to first-generation EGFR inhibitors and osimeritinib upon acquisition of T790M
Source: BMC Cancer. 2021 Nov 13;21:1215. doi: 10.1186/s12885-021-08942-x (PMC8590339; doi:10.1186/s12885-021-08942-x)

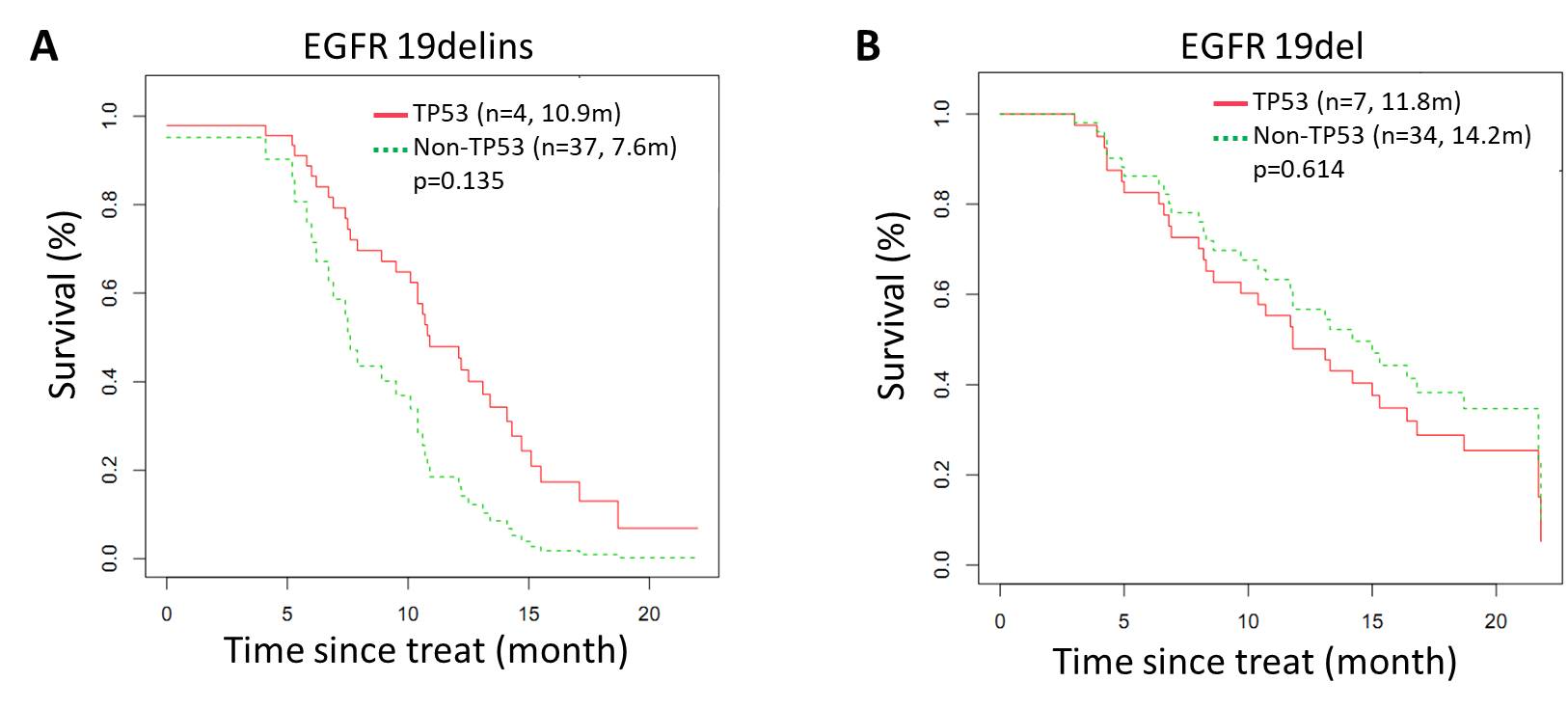

Supplement: Supplementary file 1 — Additional file 1: Figure S1. The effects of TP53 co-mutation on the survival of EGFR 19delins (A) and EGFR 19del (B) cohort. [file 12885_2021_8942_MOESM1_ESM.jpg]

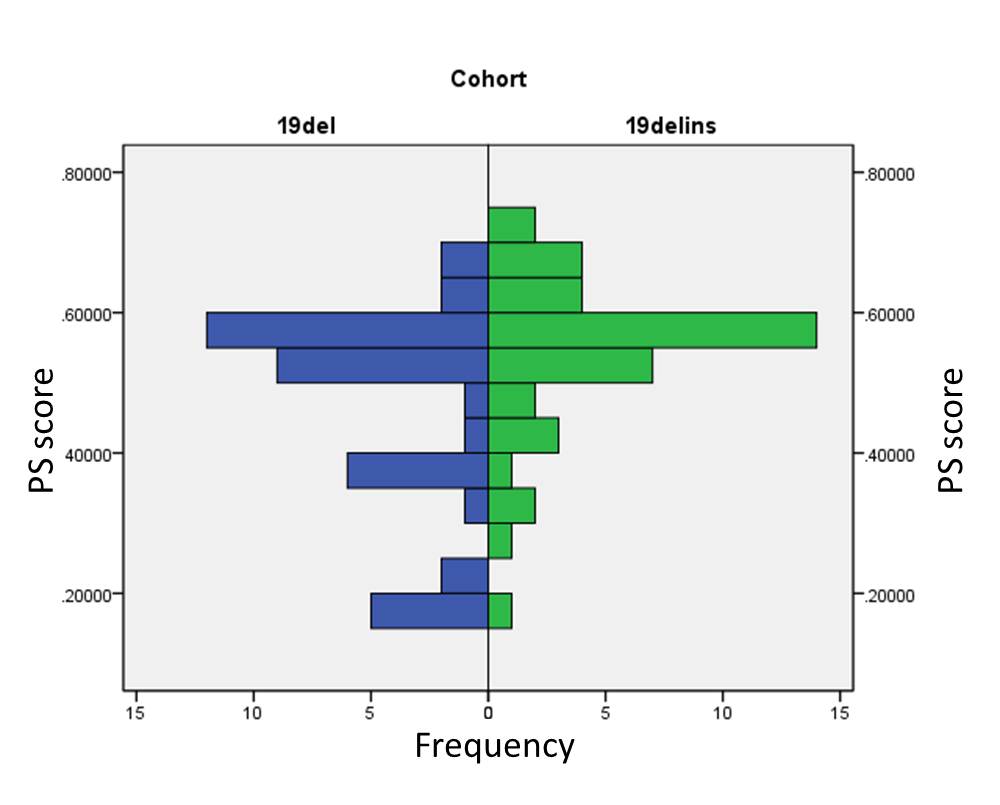

Supplement: Supplementary file 2 — Additional file 2: Figure S2. The distribution of propensity score (PS) between the cohorts of EGFR 19delins and EGFR 19del. X-axis of the population pyramid is the number of patients, and Y-axis is different propensity scores (0 ~ 1). It can be seen that propensity scores of EGFR 19delins (green) are similar to EGFR 19del (blue), mainly distributed between 0.4 and 0.6. The population pyramid is symmetric. [file 12885_2021_8942_MOESM2_ESM.jpg]
