# Supplementary material for: Patients harboring uncommon EGFR exon 19 deletion-insertion mutations respond well to first-generation EGFR inhibitors and osimeritinib upon acquisition of T790M
Source: BMC Cancer. 2021 Nov 13;21:1215. doi: 10.1186/s12885-021-08942-x (PMC8590339; doi:10.1186/s12885-021-08942-x)
Supplement: Supplementary file 3 — Additional file 3: Table S1. The VAF of EGFR 19delins mutation and EGFR 19del mutation, and the VAF of their T790M mutation acquired at progression. [file 12885_2021_8942_MOESM3_ESM.docx]

| **EGFR 19delins group** | | | **EGFR 19del group** | | |
| --- | --- | --- | --- | --- | --- |
| vairants | VAF (%) | T790M VAF (%) | vairants | VAF (%) | T790M VAF (%) |
| p.L747-T751delinsAI | 17.3 | 15.1 | p.L747_S752del | 0.53 | 0.24 |
| p.L747-A750delinsP | 31.7 | NA | p.E746_A750del | 34.4 | NA |
| p.E746_S752delinsD | 0.08 | NA | p.E746_A750del | 10.9 | 7.3 |
| p.L747_P753delinsS | 37.9 | 31 | p.E746_A750del | 46.9 | NA |
| p.L747_A750delinsP | 34.3 | NA | p.E746_A750del | 38.5 | 7.3 |
| p.L747_K754delinsA | 27.9 | NA | p.E746_A750del | 4.05 | 0.45 |
| p.E746_P753delinsLS | 47.4 | NA | p.E746_A750del | 26.3 | NA |
| p.L747_K754delinsSRE | 8.86 | NA | p.E746_A750del | 28.2 | NA |
| p.E746_S752delinsV | 39.6 | NA | p.E746_A750del | 1.63 | NA |
| p.L747_S752delinsQ | 64.6 | 31.1 | p.E746_A750del | 29.3 | NA |
| p.L747_P753delinsS | 10.7 | NA | p.E746_A750del | 18.4 | NA |
| p.L747_T751delinsP | 50.6 | NA | p.E746_A750del | 58.2 | 10.2 |
| p.E746_T751delinsI | 56 | NA | p.E746_A750del | 14.5 | NA |
| p.L747_P753delinsS | 13.81 | NA | p.E746_A750del | 46.7 | 3.5 |
| p.L747_A755delinsSKG | 44.4 | NA | p.E746_A750del | 47.9 | 7.6 |
| p.L747_P753delinsS | 34.2 | 17.3 | p.E746_A750del | 29.7 | 15 |
| p.L747_P753delinsS | 6.77 | NA | p.E746_A750del | 17.4 | 4.7 |
| p.L747_P753delinsS | 62.5 | NA | p.E746_A750del | 37 | NA |
| p.L747_A750delinsP | 10.9 | NA | p.E746_A750del | 14.9 | NA |
| p.L747_P753delinsS | 28.4 | NA | p.E746_A750del | 32 | 6.1 |
| p.L747_T751delinsP | 37 | NA | p.L747_S752del | 40.8 | NA |
| p.T751_I759delinsN | 15.3 | NA | p.E746_A750del | 4.89 | NA |
| p.L747_P753delinsS | 47.5 | NA | p.E746_A750del | 35.4 | NA |
| p.A750_I759delinsPS | 61.1 | NA | p.E746_A750del | 15.7 | NA |
| p.L747_P753delinsS | 32.1 | NA | p.E746_A750del | 6.9 | NA |
| p.E746_S752delinsV | 45.4 | NA | p.E746_A750del | 36.2 | NA |
| p.L747_A750delinsP | 40.2 | NA | P.L747_T751del | 30.1 | 0.03 |
| p.L747_T751delinsP | 23.7 | 13.6 | p.E746_A750del | 27.9 | NA |
| p.L747_T751delinsP | 35.3 | NA | p.E746_A750del | 27.7 | 7.3 |
| p.E746_A750delinsIP | 50.9 | 23 | p.E746_A750del | 63 | NA |
| p.L747_P753delinsS | 23.9 | NA | p.E746_A750del | 18.8 | 0.3 |
| p.L747_A750delinsP | 25.8 | 0.94 | p.L747_S752del | 25.6 | NA |
| p.L747_A750delinsP | 16.3 | 7.4 | p.E746_A750del | 85.2 | NA |
| p.L747_A750delinsP | 19.3 | NA | p.E746_A750del | 8.37 | NA |
| p.L747_T751delinsP | 6.4 | NA | p.E746_A750del | 86.4 | 21.1 |
| p.L747_A750delinsP | 15.8 | 0.88 | p.E746_A750del | 39.7 | NA |
| p.L747_A750delinsP | 22.5 | 6.3 | P.L747_T751del | 11.4 | NA |
| p.E746_S752delinsV | 15.4 | NA | p.E746_A750del | 74.8 | NA |
| p.L747_T751delinsP | 33.9 | 4.2 | p.E746_A750del | 67.1 | NA |
| p.L747_K754delinsSR | 90.8 | 23.1 | p.E746_A750del | 75.4 | 47.6 |
| p.L747_S752delinsQH | 16.6 | NA | p.E746_A750del | 41.9 | NA |

Table S1 The VAF of EGFR 19delins mutation and EGFR 19del mutation, and the VAF of their T790M mutation acquired at progression.

19delins, EGFR exon 19 deletion-insertion; 19del, EGFR exon 19 deletion; VAF, variant allele frequency; NA, not applicable.
